# Alternative exon splicing and differential expression in pancreatic islets reveals candidate genes and pathways implicated in early diabetes development

**DOI:** 10.1007/s00335-021-09869-1

**Published:** 2021-04-20

**Authors:** Sayeed ur Rehman, Tanja Schallschmidt, Axel Rasche, Birgit Knebel, Torben Stermann, Delsi Altenhofen, Ralf Herwig, Annette Schürmann, Alexandra Chadt, Hadi Al-Hasani

**Affiliations:** 1 Institute for Clinical Biochemistry and Pathobiochemistry, German Diabetes Center (DDZ), Leibniz Center for Diabetes Research at Heinrich Heine University, Medical Faculty, Duesseldorf, Germany; 2 German Center for Diabetes Research (DZD), München-Neuherberg, Germany; 3 Department of Computational Molecular Biology, Max Planck Institute for Molecular Genetics, Berlin, Germany; 4 German Institute of Human Nutrition, Potsdam, Germany; 5 Present Address: Department of Biochemistry, School of Chemical and Life Sciences, Jamia Hamdard, New Delhi, 110062 India

## Abstract

Type 2 diabetes (T2D) has a strong genetic component. Most of the gene variants driving the pathogenesis of T2D seem to target pancreatic β-cell function. To identify novel gene variants acting at early stage of the disease, we analyzed whole transcriptome data to identify differential expression (DE) and alternative exon splicing (AS) transcripts in pancreatic islets collected from two metabolically diverse mouse strains at 6 weeks of age after three weeks of high-fat-diet intervention. Our analysis revealed 1218 DE and 436 AS genes in islets from NZO/Hl vs C3HeB/FeJ. Whereas some of the revealed genes present well-established markers for β-cell failure, such as *Cd36* or *Aldh1a3*, we identified numerous DE/AS genes that have not been described in context with β-cell function before. The gene *Lgals2*, previously associated with human T2D development, was DE as well as AS and localizes in a quantitative trait locus (QTL) for blood glucose on Chr.15 that we reported recently in our N_2_(NZOxC3H) population. In addition, pathway enrichment analysis of DE and AS genes showed an overlap of only half of the revealed pathways, indicating that DE and AS in large parts influence different pathways in T2D development. PPARG and adipogenesis pathways, two well-established metabolic pathways, were overrepresented for both DE and AS genes, probably as an adaptive mechanism to cope for increased cellular stress. Our results provide guidance for the identification of novel T2D candidate genes and demonstrate the presence of numerous AS transcripts possibly involved in islet function and maintenance of glucose homeostasis.

**Supplementary Information:**

The online version contains supplementary material available at 10.1007/s00335-021-09869-1.

## Introduction

The pathogenesis of type 2 diabetes (T2D), which is characterized by chronically elevated blood glucose levels, is closely linked with obesity. This is illustrated by the fact that approximately 90% of all patients with T2D are obese (CDC 2020). Obesity typically induces insulin resistance of the insulin-responsive peripheral organs which forces the pancreatic β-cells to increase their insulin secretion (Simonis-Bik et al. 2009). When the demand for insulin exceeds the capacity of the β-cells to secrete sufficient insulin, the uptake of glucose in the peripheral organs becomes impaired and T2D becomes manifest. While environmental factors, such as diet and physical activity, seem to play more important role in the development of insulin resistance, the ability of the β-cells to boost their insulin secretory capacity is assumed to be predominantly driven by genetic factors (Thomsen et al. 2016).

Until today, most of the genetic variants as well as the underlying regulatory mechanisms responsible for the wide range in T2D susceptibility in humans remain to be elucidated (Morris et al. 2012; Tsaih et al. 2014). Inbred mouse strains that widely differ in their susceptibility to develop obesity and T2D provide genetic diversity with respect to diabetes risk, similar as observed in the human population. However, mouse models offer substantial advantages over human studies. In contrast to humans, mice can be used for the engineering of gene mutations with well-established molecular genetic tools and tissues and be collected for all kind of analyses (Attie et al. 2017; Kleinert et al. 2018). Moreover, in animal studies, the environment, such as the diet, can be strictly controlled, and thus, mouse models have become indispensable for the identification and molecular characterization of novel “diabetes genes” in humans.

The New Zealand Obese (NZO) mouse strain represents an established mouse model for polygenetic obesity and T2D as it develops all features of the human disease in response to a high-fat diet (HFD), indicating that the causal gene variants are similar as in humans (Joost 2010; Joost and Schurmann 2014). Upon HFD, approximately 50% of the NZO males progress from obesity and insulin resistance into severe β-cell failure and overt T2D (Lange et al. 2006). In contrast, C3H mice are able to compensate for high-fat-diet feeding which is likely due to their robust insulin secretory capacity (Kaku et al. 1988; Schallschmidt et al. 2018; Toye et al. 2005).

In the past, we (Chadt et al. 2008; Schallschmidt et al. 2018; Scherneck et al. 2009; Schwerbel et al. 2020; Vogel et al. 2012, 2018) and others (Andrikopoulos et al. 2016; Leiter et al. 1998) have used the NZO strain in genome-wide linkage studies attempting to identify novel quantitative trait loci (QTL) and the underlying causal gene variants that may contribute to the high T2D susceptibility.

In all these studies, differential gene expression (DE) was used as major criteria for the nomination of candidate genes, which has been successful for some of the loci (Andrikopoulos et al. 2016; Chadt et al. 2008; Scherneck et al. 2009; Schwerbel et al. 2020; Vogel et al. 2012). However, the detection of candidate genes based on DE is limited as it will not survey genes whose functions may be changed due to post-transcriptional modifications. It is well established that mRNA processing can influence features like binding properties, intracellular localization, enzymatic activity, or protein stability. The resulting functional impacts can be diverse, ranging from imperceptible consequences to a complete loss of function.

Alternative Splicing (AS), a post-transcriptional molecular process which generates multiple mRNA transcripts from a single gene template and thus structurally and functionally different protein isoforms, has gained increasing interest in context with the discovery of novel risk genes for human diseases (Novoyatleva et al. 2006; Stoilov et al. 2002) including T2D (Pihlajamaki et al. 2011). About 94% of the human genome is alternatively spliced (Ward and Cooper 2010) and it is further estimated that half of all disease-causing mutations affect splicing (Lopez-Bigas et al. 2005; Pan et al. 2008). The different splice isoforms often appear tissue-specifically expressed (Bland et al. 2010), where they function in the differentiation and development of the organs (Nilsen and Graveley 2010). The ability of the organism to generate different splice isoforms can be viewed as an adaptation process allowing to respond to distinct developmental and metabolic cues (Nilsen and Graveley 2010; Salomonis et al. 2010). Consistent with this assumption, many key regulator genes of pancreatic β-cell function, including NO1, HNF-1a, IPF-1, GCK, SUR1, TCF7L2, VEGF, and NOVA1, are known to be regulated by alternative splicing in humans (Dlamini et al. 2017).

In the presents study, we analyzed exon array data from pancreatic islets collected from T2D-prone NZO/Hl and T2D-resistant C3HeB/FeJ mice and fed with a high-fat diet with 45% calories from fat for three weeks. This analysis revealed DE as well as AS genes between the two strains that may contribute to their different T2D susceptibilities and, thus, represent novel candidate genes involved in early stages of T2D development. Using further bioinformatic tools, we performed pathway enrichment, gene network, as well as gene ontology enrichment analysis of the DE and AS genes.

## Materials and methods

### Experimental animals

Experiments involving mice were approved by the Ethics Committee of the State Ministry of Agriculture, Nutrition, and Forestry, State of North Rhine-Westphalia, Germany (references: 84-02.04.2013.A118). Diabetes-prone NZO/Hl and diabetes-resistant C3HeB/FeJ mice (Schallschmidt et al. 2018) were housed at three to six mice per cage (Macrolon type III) at a constant temperature of 22° and a 12 h light–dark cycle (lights on at 6 am). Animals had ad libitum access to food and water*.* After weaning at the age of 21 days, all experimental animals received a HFD containing 45 kcal% fat, 20 kcal% protein, and 35 kcal% carbohydrates with 4.73 kcal/gm energy (D12451; Research Diets, New Brunswick, NJ).

### Determination of blood glucose, body fat, and cumulative T2D prevalence

Blood glucose was measured every week using CONTOUR® XT glucometer (Bayer Consumer Care AG, Leverkusen, Germany). An electronic scale (Sartorius, Gottingen, Germany) was used to measure the body weight, while non-invasive nuclear magnetic resonance spectroscopy (EchoMRI™-100 system, Echo Medical Systems, Houston, USA) was used to study the body composition of the mice every week. T2D prevalence was calculated by determining the cumulative number of diabetic mice (blood glucose > 300 mg/dl for at least three consecutive weeks) and expressing the percentage of affected mice in relation to the total number of mice.

### Analysis of plasma insulin

Plasma insulin levels from 6-week-old mice were measured by ELISA (Insulin: Mouse Ultrasensitive ELISA Kit, DRG instruments GmbH) using the manufacturer’s protocol. Blood was obtained by cardiac puncture after 6 h of overnight fasting.

### Sacrificing of mice and pancreatic islet isolation

After 3 weeks of HFD intervention, mice were sacrificed by cervical dislocation at 6 weeks of age, and the pancreatic islets were isolated by ductal collagenase perfusion of the pancreas following a protocol described previously (Yesil et al. 2009). The isolated islets were regenerated overnight by incubation in CMRL medium (Connaught Medical Research Laboratories) at 37 °C with 5% CO_2_.

### RNA extraction and microarray analysis

Regardless of the islet size, approximately 150 pancreatic islets for each sample were used for RNA extraction using RNeasy mini kit (QIAGEN, Hilden, Germany) according to the manufacturer’s protocol. The quality of isolated RNA was assessed using Agilent 2100 Bioanalyzer (Agilent Technologies, Santa Clara, CA, USA). Samples with RNA Integrity Number (RIN) values > 8 were processed further for microarray analysis (*n* = 5 per genotype) using Affymetrix-Chip (GeneChip® Mouse Gene 1.0 ST Array) as previously described (Schallschmidt et al. 2018).

### cDNA synthesis and quantitative real-time (qRT) PCR

cDNA was synthesized using GoScript™ Reverse Transcriptase Kit (Promega, Madison, USA) using 500 ng RNA. For qRT-PCR, the GoTaq® qPCR Master Mix (Promega, Madison, USA) on a QuantStudio 7 Flex PCR System (Applied Biosystems, Foster City, USA) was used. *TATA box binding protein* (*Tbp*) was used as an endogenous control and gene expression was quantified using the 2^−ΔΔCT^ method (Livak and Schmittgen 2001).

### Differential expression and alternative splicing analysis

Oligo probe sequences were mapped to Ensembl exons (release 74) using customCDF tools as described previously (Dai et al. 2005). Exons were mapped to 21,406 genes via BioMart (Durinck et al. 2009). Intra-chip normalization was performed via MAT from Johnson and colleagues (Johnson et al. 2006) including GC correction. For inter-chip normalization, quantile normalization was used. Computations were run in R/BioConductor in version 3.0.2/2.13, respectively (Gentleman et al. 2004).

For gene expression analysis, exons were agglomerated to genes, and the median of all probe values annotated for a specific gene was used as expression estimate in each condition replicate (NZO and C3H). Additionally, gene expression over background was judged by comparing the probe values to the 75%-quantile of background control probe values with the same GC sequence content, and a gene was called "present" when more than 50% of its exon probes were above this threshold. Differentially expressed genes were selected by three criteria: (1) The gene was called "present" in at least one of two conditions (NZO and C3H); (2) Gene expression has a ratio of $\ge$ 1.33 or $\le$ 0.75 between the two mouse strains as well as (3) the two-sided Wilcoxon test between gene expression values of the replicates (*n* = 5) is significant with a *p*-value $\le$ 0.05.

For alternative splicing analysis, an AS robust prediction method based on entropy (ARH) was used (Rasche and Herwig 2010). ARH is based on a robust entropy model based on the exon expression ratios with respect to two experimental conditions. A deviation in exons leads to a dominating effect on the entropy and a high ARH value. Exon probe values were used directly for each gene and a splicing prediction factor was computed based on the deviation of the log2-ratio of the exon expression in the two conditions (NZO and C3H) from the median log2-ratio over all exons. These splicing deviations are then transformed into a splicing probability distribution that is subsequently evaluated using entropy. High entropy values indicate genes with spliced exons between the two experimental conditions. Exon splicing events with an ARH *p*-value $\le$ 0.05 were considered significant.

### Pathway enrichment and gene ontology analysis

The enriched pathways associated with the DE and AS genes were analyzed using Ingenuity Pathway Analysis (IPA), DAVID (https://david.ncifcrf.gov/home.jsp) and InnateDB (http://www.innatedb.com/). Gene network analysis was performed using IPA, while Gene Ontology (GO) analysis was performed using the InnateDB tool.

## Results

### NZO mice develop early onset obesity and hyperglycemia

**Fig. 1:**
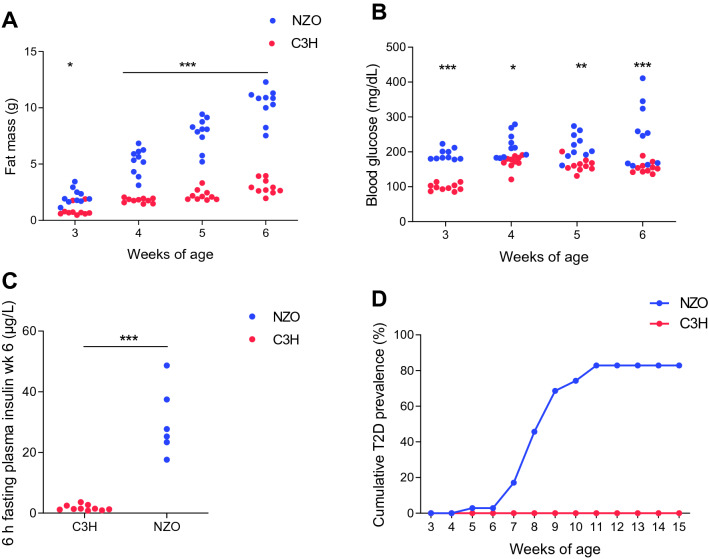
Metabolic features of NZO and C3H mice. After weaning at 3 weeks of age, Fat mass (**a**) and blood glucose levels (**b**) were measured weekly until week 6 of age. Body composition was measured via non-invasive nuclear magnetic resonance spectroscopy. Plasma insulin levels (**c**) were measured via ELISA after 6 h of fasting at week 6 of age. For the determination of the cumulative T2D prevalence (**d**) a different cohort of NZO and C3H mice was used which were sacrificed at adult stage of age. Dots represent single male animals or mean values (C3H: red dots, NZO: blue dots; **a**–**c**: *n* = 10, **d**: *n* = 29–35). Statistical differences between strains were calculated by two-way ANOVA followed by post hoc Bonferroni test (**a** and **b**) or by unpaired *t*-test (**c**). *P, 0.05, **P, 0.01 and ***P, 0.001 by comparison to NZO. *NZO* New Zealand Obese; *wk* week

For our study, we used two metabolically divergent inbred mouse strains, T2D-prone NZO mice and T2D-resistant C3H animals. We validated the different T2D susceptibilities by phenotyping the animals after weaning for three weeks on HFD for basic metabolic features. NZO mice gained substantially more body weight with the duration of the HFD, which was mainly due to a difference in fat mass (week 6 of age: 10.34 ± 0.4 g vs. 2.96 ± 0.2 g, *P* < 0.001; Fig. 1a). Moreover, already at 3 weeks of age, on the day of the start of the HFD intervention, NZO animals exhibited significantly higher blood glucose levels compared to C3H mice (192 ± 5 mg/dl vs. 99 ± 3 mg/dl, *P* < 0.001; Fig. 1b). These differences in glycemia were also evident during HFD feeding (week 6 of age: 250 ± 26 mg/dl vs. 155 ± 5 mg/dl, *P* < 0.001). In addition, 6 h fasting plasma insulin levels at 6 weeks of age were almost 20-fold higher in the obese strain (30.0 ± 4.2 µg/l vs. 1.7 ± 0.3 µg/l, *P* < 0.001; Fig. 1c). Phenotyping of a different cohort of NZO and C3H mice, which were monitored until week 15 of age, showed that about half of all NZO mice become diabetic at the age of 8 weeks, reaching a maximal T2D prevalence with 83% at 11 weeks of age. In contrast, none of the C3H mice developed T2D (Fig. 1d).

### Microarray analysis reveals 1218 DE and 436 AS genes in islets from NZO vs C3H

After three weeks of HFD intervention, transcriptional profiling in the pancreatic islets collected from 6 weeks old C3H and NZO mice was conducted using exon microarray analysis. Our analysis revealed 1218 genes being differentially expressed between the pancreatic islets from C3H and NZO. While 753 genes were found to be upregulated in NZO, 465 genes were overexpressed in C3H (Supplementary File 1). A list of the top genes (log2 C3H/NZO < − 1.5) overexpressed in NZO is shown in Table 1a, whereas Table 1b includes the top genes (log2 C3H/NZO > 1.2) overexpressed in C3H. In total, 14 DE genes were associated with mitochondrial function (Table 2). For validation of microarray data, 22 DE genes were selected for qPCR analysis in pancreatic islets between NZO vs C3H. Differential expression of 20 genes was validated (Supplementary File 8), providing evidence for the reliability of our microarray results.

**Table 1a:** Top genes (log2 <  − 1.5) overexpressed in pancreatic Islets from NZO vs C3H

Gene	Log2 [C3H/NZO]	Description
*Reg3b*	− 3.94	Regenerating islet-derived 3 beta
*1810009J06Rik*	− 3.9	RIKEN cDNA 1810009J06 gene
*Prss3*	− 3.8	Protease, serine, 3
*Spink3*	− 3.65	Serine peptidase inhibitor, Kazal type 3
*Cpb2*	− 3.53	Carboxypeptidase B2 (plasma)
*Try4*	− 3.51	Trypsin 4
*Try5*	− 3.37	Trypsin 5
*Cldn11*	− 3.31	Claudin 11
*Kcnip3*	− 3.29	Kv channel interacting protein 3, calsenilin
*Gm5771*	− 3.28	Predicted gene 5771
*Aldh1a3*	− 2.83	Aldehyde dehydrogenase family 1, subfamily A3
*Try10*	− 2.79	Trypsin 10
*Reg2*	− 2.77	Regenerating islet-derived 2
*Pigr*	− 2.7	Polymeric immunoglobulin receptor
*Lgals2*	− 2.68	Lectin, galactose-binding, soluble 2
*Reg1*	− 2.64	Regenerating islet-derived 1
*Cela3b*	− 2.6	Chymotrypsin-like elastase family, member 3B
*Pnlip*	− 2.47	Pancreatic lipase
*Csf2rb2*	− 2.45	Colony stimulating factor 2 receptor, beta 2, low affinity (granulocyte–macrophage)
*Cuzd1*	− 2.42	CUB and zona pellucida-like domains 1
*Klk1b5*	− 2.42	Kallikrein 1-related peptidase b5
*Muc13*	− 2.39	Mucin 13, epithelial transmembrane
*Pla2g1b*	− 2.36	Phospholipase A2, group IB, pancreas
*Cela2a*	− 2.35	Chymotrypsin-like elastase family, member 2A
*Gp2*	− 2.31	Glycoprotein 2 (zymogen granule membrane)
*Klk1*	− 2.25	Kallikrein 1
*Sycn*	− 2.16	Syncollin
*Gm10334*	− 2.15	Predicted gene 10334
*Gm4744*	− 2.1	Predicted gene 4744
*Hbb-bs*	− 2.08	Hemoglobin, beta adult s chain
*Ank2*	− 2	Ankyrin 2, brain
*Ctse*	− 2	Cathepsin E
*Ctrl*	− 1.92	Chymotrypsin-like
*Zg16*	− 1.92	Zymogen granule protein 16
*Pf4*	− 1.92	Platelet factor 4
*2210010C04Rik*	− 1.91	RIKEN cDNA 2210010C04 gene
*Pdia2*	− 1.91	Protein disulfide isomerase associated 2
*Fgg*	− 1.91	Fibrinogen gamma chain
*Cpb1*	− 1.86	Carboxypeptidase B1 (tissue)
*Cpa2*	− 1.85	Carboxypeptidase A2, pancreatic
*Cck*	− 1.85	Cholecystokinin
*Rnase1*	− 1.81	Ribonuclease, RNase A family, 1 (pancreatic)
*Mx1*	− 1.8	Myxovirus (influenza virus) resistance 1
*Gjb4*	− 1.79	Gap junction protein, beta 4
*Klk1b4*	− 1.79	Kallikrein 1-related pepidase b4
*Pnliprp1*	− 1.76	Pancreatic lipase-related protein 1
*Cel*	− 1.73	Carboxyl ester lipase
*Selp*	− 1.73	Selectin, platelet
*Prss2*	− 1.7	Protease, serine, 2
*Cd36*	− 1.69	CD36 antigen
*Prrg4*	− 1.67	Proline rich Gla (G-carboxyglutamic acid) 4 (transmembrane)
*Serpinb1a*	− 1.67	Serine (or cysteine) peptidase inhibitor, clade B, member 1a
*Muc1*	− 1.66	Mucin 1, transmembrane
*Fgl1*	− 1.62	Fibrinogen-like protein 1
*Ctsh*	− 1.59	Cathepsin H
*Pnliprp2*	− 1.58	Pancreatic lipase-related protein 2
*Clps*	− 1.56	Colipase, pancreatic
*Reg3g*	− 1.54	Regenerating islet-derived 3 gamma
*Tmc7*	− 1.53	Transmembrane channel-like gene family 7

**Table 1b:** Top genes (log2 > 1.2) overexpressed in pancreatic Islets from C3H vs NZO

Gene	Log2 [C3H/NZO]	Description
*Klk1b22*	2.89	Kallikrein 1-related peptidase b22
*Gm10288*	2.65	Predicted gene 10288
*Pianp*	2.54	PILR alpha associated neural protein
*Rab3c*	2.49	RAB3C, member RAS oncogene family
*Akr1c12*	2.12	Aldo–keto reductase family 1, member C12
*Pcp4*	2.06	Purkinje cell protein 4
*Nefm*	2.05	Neurofilament, medium polypeptide
*Atp4a*	1.83	ATPase, H+/K+ exchanging, gastric, alpha polypeptide
*mt-Tf*	1.78	Mitochondrially encoded tRNA phenylalanine
*Rnf7*	1.62	Ring finger protein 7
*Slc6a17*	1.61	Solute carrier family 6 (neurotransmitter transporter), member 17
*4933431E20Rik*	1.56	RIKEN cDNA 4933431E20 gene
*Trnp1*	1.56	TMF1-regulated nuclear protein 1
*Gm8840*	1.53	Predicted gene 8840
*Tac1*	1.52	Tachykinin 1
*Nrxn1*	1.51	Neurexin I
*Lipo1*	1.49	Lipase, member O1
*Gm15698*	1.49	Predicted gene 15698
*Angptl7*	1.43	Angiopoietin-like 7
*Vip*	1.35	Vasoactive intestinal polypeptide
*2610305D13Rik*	1.34	RIKEN cDNA 2610305D13 gene
*Fam163a*	1.34	Family with sequence similarity 163, member A
*Galr1*	1.3	Galanin receptor 1
*Calb1*	1.28	Calbindin 1
*Gm26335*	1.26	Predicted gene, 26335
*Gria2*	1.26	Glutamate receptor, ionotropic, AMPA2 (alpha-2)
*Tubb4a*	1.25	Tubulin, beta 4A class IVA
*Rims1*	1.24	Regulating synaptic membrane exocytosis 1
*Orc3*	1.23	Origin recognition complex, subunit 3
*Mansc1*	1.22	MANSC domain containing 1
*Arpc5*	1.22	Actin related protein 2/3 complex, subunit 5
*St8sia3*	1.21	ST8 alpha-*N*-acetyl-neuraminide alpha-2,8-sialyltransferase 3
*Slc2a2*	1.21	Solute carrier family 2 (facilitated glucose transporter), member 2
*Gad1*	1.21	Glutamate decarboxylase 1
*Ddc*	1.21	Dopa decarboxylase
*Ncam2*	1.21	Neural cell adhesion molecule 2

**Table 2:** List of differentially expressed mitochondrial-associated genes in C3H vs. NZO pancreatic islets

Gene	Description	log2 [C3H/NZO]
*Overexpressed in NZO vs. C3H*		
*Acaa2*	Acetyl-Coenzyme A acyltransferase 2 (mitochondrial 3-oxoacyl-Coenzyme A thiolase)	− 0.549
*Mrpl35*	Mitochondrial ribosomal protein L35	− 0.911
*Oxsm*	3-Oxoacyl-ACP synthase, mitochondrial	− 0.462
*Slc25a24*	Solute carrier family 25 (mitochondrial carrier, phosphate carrier), member 24	− 0.539
*Overexpressed in C3H vs. NZO*		
*Mrpl53*	Mitochondrial ribosomal protein L53	0.494
*Mrps10*	Mitochondrial ribosomal protein S10	0.606
*mt-Tf*	Mitochondrially encoded tRNA phenylalanine	1.78
*mt-Ti*	Mitochondrially encoded tRNA isoleucine	0.89
*mt-Tl2*	Mitochondrially encoded tRNA leucine 2	0.48
*mt-Tm*	Mitochondrially encoded tRNA methionine	0.79
*mt-Tq*	Mitochondrially encoded tRNA glutamine	0.564
*Slc25a17*	Solute carrier family 25 (mitochondrial carrier, peroxisomal membrane protein), member 17	0.997
*Slc25a19*	Solute carrier family 25 (mitochondrial thiamine pyrophosphate carrier), member 19	0.462
*Tfb1m*	Transcription factor B1, mitochondrial	0.468

In addition, events of alternative splicing were studied using ARH analysis. In total, 3480 exons corresponding to 436 different genes were found to be alternatively spliced between the two strains. 206 of these genes overlapped with the DE category. Table 3 presents the top 20 out of the 436 AS pancreatic islets genes in C3H vs. NZO. Supplementary File 2 includes all 3480 significant AS exons with the corresponding genes as well and more detailed information on each AS event, such as the exon position.

**Table 3:** List of top 20 AS pancreatic islets genes in C3H vs. NZO

Gene ID	Description	ARH	ARH *p-*value	Number of AS exons
*Apobec1*	Apolipoprotein B mRNA editing enzyme, catalytic polypeptide 1	2	5.30E−07	12
*Kcnip3*	Kv channel interacting protein 3, calsenilin	1.9	6.80E−07	5
*Nrip1*	Nuclear receptor interacting protein 1	1.8	7.80E−07	13
*Ermard*	ER membrane associated RNA degradation	1.7	8.70E−07	6
*Rps3*	Ribosomal protein S3	1.4	1.50E−06	8
*Nop56*	NOP56 ribonucleoprotein	1.1	3.00E−06	16
*H2-D1*	Histocompatibility 2, D region locus 1	0.9	5.80E−06	6
*Gstm2*	Glutathione S-transferase, mu 2	0.9	6.00E−06	3
*Zfp715*	Zinc finger protein 715	0.75	1.00E−05	8
*Lefty1*	Left right determination factor 1	0.75	1.00E−05	3
*Cr1l*	Complement component (3b/4b) receptor 1-like	0.67	1.40E−05	3
*Lgals2*	Lectin, galactose-binding, soluble 2	0.64	1.60E−05	3
*Zfp110*	Zinc finger protein 110	0.64	1.70E−05	9
*Pianp*	PILR alpha associated neural protein	0.62	1.80E−05	8
*Fkbp3*	FK506 binding protein 3	0.61	1.80E−05	6
*Tom1l1*	Target of myb1-like 1 (chicken)	0.6	1.90E−05	5
*Eapp*	E2F-associated phosphoprotein	0.6	2.00E−05	15
*Rnf14*	Ring finger protein 14	0.58	2.20E−05	7
*Srsf10*	Serine/arginine-rich splicing factor 10	0.57	2.20E−05	3
*Trpm1*	Transient receptor potential cation channel, subfamily M, member 1	0.55	2.50E−05	10

All significant 3480 AS exons as well as detailed information on the AS event, including the exact exon position, are show in Supplementary File 2

### Pathway enrichment analysis of DE genes

**Fig. 2:**
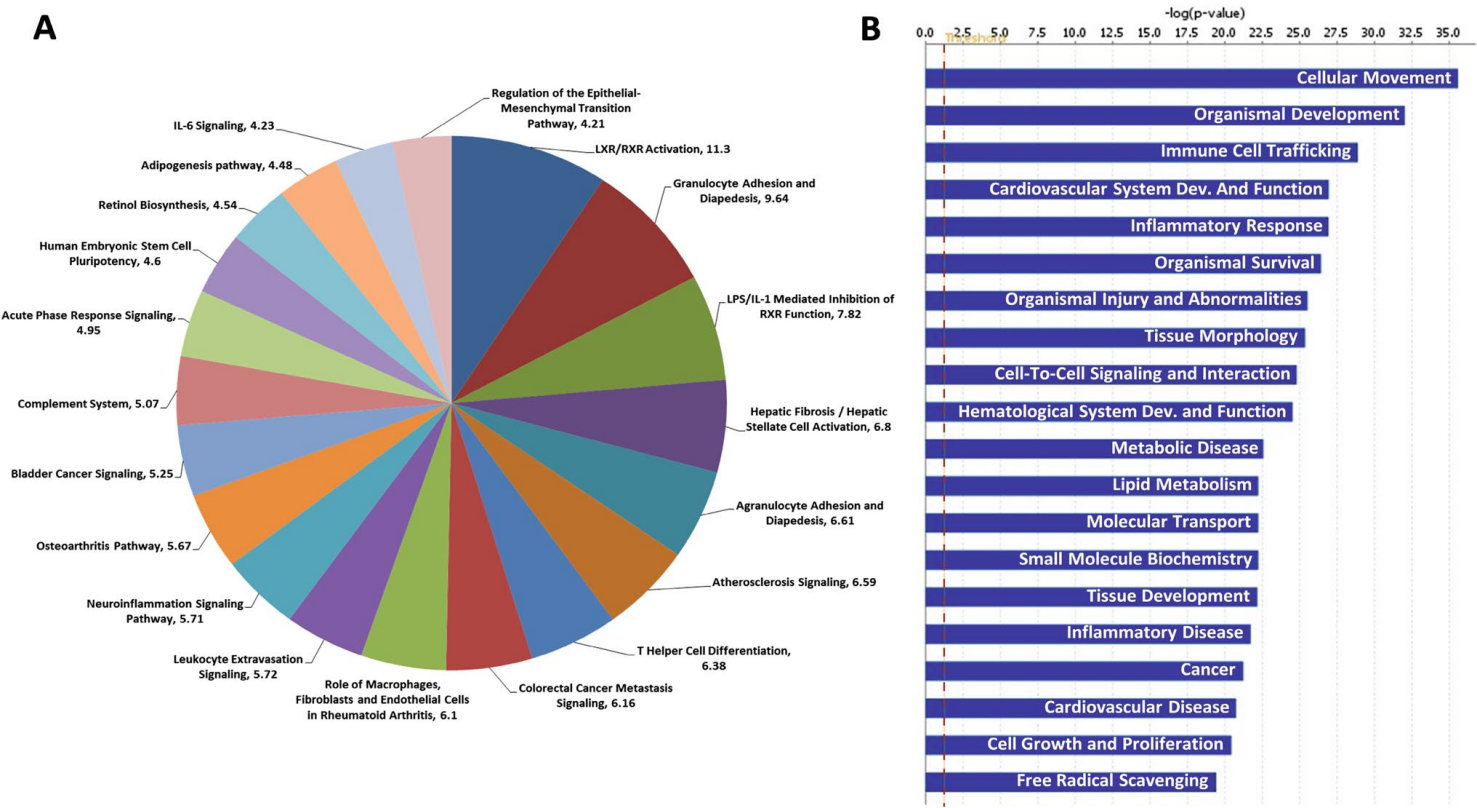
Ingenuity Pathway Analysis of differentially expressed (DE) genes of pancreatic islets between C3H vs. NZO mice. The list of DE genes was obtained after processing the DNA microarray data. **a** Top canonical pathways overrepresented in DE genes. Statistical significance is mentioned along with the pathway name as − log(*p*-value). **b** Top disease and function overrepresented by DE genes. The red dotted line represents the significance threshold (*p* < 0.05). Only top disease and function categories are mentioned

All DE genes were further analyzed for significantly enriched pathways and networks using Ingenuity Pathway Analysis (IPA) tool. In total, 149 canonical pathways were significantly overrepresented (Supplementary File 3), and the most significant ones are shown in Fig. 2a. The top five pathways are LXR/RXR Activation (*p*-value = 5.22E−12), Granulocyte Adhesion and Diapedesis (*p*-value = 2.27E−10), LPS/IL-1-Mediated Inhibition of RXR Function (*p*-value = 1.52E−08), Hepatic Fibrosis/Hepatic Stellate Cell Activation (*p*-value = 1.58E−07), and Agranulocyte Adhesion and Diapedesis (*p*-value = 2.48E−07).

We further searched for diseases and functions overrepresented by the DE genes, and 68 categories were found to be represented significantly (Supplementary File 4). Figure 2b represents the top diseases and functions from our list that includes cellular movement, organismal development, immune cell trafficking, cardiovascular system development and function, and inflammatory response. Also, the top diseases and disorders overrepresented by DE genes include inflammatory response (*p-*value = 1.05E−08 − 1.13E−27), organismal injury and abnormalities (*p-*value = 1.03E−08 − 2.85E−26), metabolic disease (*p-*value = 9.46E−09 − 2.54E−23), inflammatory disease (*p-*value = 5.44E−09 − 1.81E−22), and cancer (*p-*value = 9.57E−09 – 5.53E−22).

**Fig. 3:**
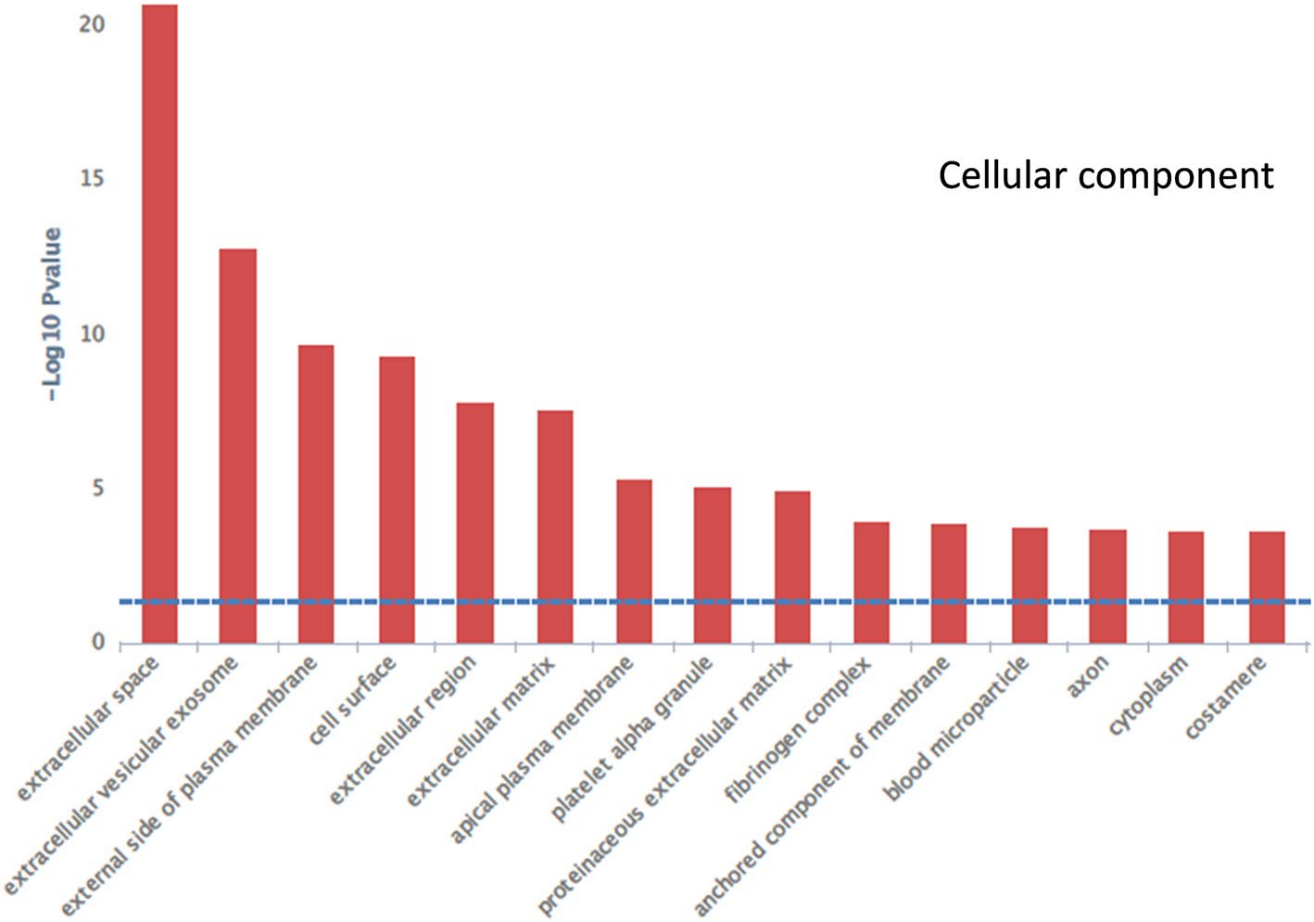
GO analysis using InnateDB tool. List of DE genes was uploaded in the InnateDB portal to study the cellular components overrepresented in DE genes. Only top components are shown in this figure while the complete list is provided in Supplementary File 5. The blue line represents the threshold value corresponding to a *p*-value of 0.05

Furthermore, to investigate the cellular component related to our DE genes, we conducted Gene Ontology (GO) analysis. In total, 148 cellular components were identified to be differentially regulated between NZO and C3H islets (Supplementary File 5). As illustrated in Fig. 3, some of the top cellular components include extracellular space, extracellular vesicular exosome, external side of the plasma membrane, cell surface, extracellular region, and extracellular matrix (ECM). Out of 33 DE genes associated with the ECM, 28 were upregulated in NZO and only five revealed higher mRNA expression in C3H islets (Table 4). Moreover, several of the DE genes associated with ECM have been annotated to play roles in inflammation, diabetes, and cancer (Table 4).

**Table 4:** List of differentially expressed ECM genes in pancreatic islets from C3H vs. NZO

Gene	Role of genes in inflammation, diabetes, and cancer (reference^a^)
*Upregulated in NZO vs. C3H*	
*Adamts1*	Upregulation linked with metastatic pancreatic cancer (Masui et al. 2001)
*Adamts4*	Loss reduces high-fat diet-induced atherosclerosis (Kumar et al. 2016)
*Adamts5*	Deteriorating effect on the liver during diet‐induced obesity (Bauters et al. 2016)
*Adamts9*	T2D-associated gene (Ho et al. 2013)
*Aebp1*	Role in sex-specific diet-induced obesity (Zhang et al. 2005)
*Bgn*	Overexpression observed in obesity (Nadler et al. 2000)
*Clu*	Clusterin gene polymorphisms associated with T2D (Daimon et al. 2011)
*Col6a6*	Differentially expressed in adipose tissue with a crown-like structure (CLS) and without CLS (Lê et al. 2011)
*Crip2*	Upregulation observed in retinas of diabetic mice (Bogdanov et al. 2014)
*Cthrc1*	Highly expressed in pancreatic cancer (Lee et al. 2016)
*Dcn*	Overexpression observed in pancreatic cancer (Köninger et al. 2004)
*Ecm1*	Overexpression observed in breast cancer tissue samples (Wu et al. 2012)
*Emilin1*	Exerts a protective role on tumor growth (Danussi et al. 2012)
*Il1rl1*	Key regulator of the inflammatory process (Akhabir and Sandford 2010)
*Lgals3*	Deficiency accelerates high-fat-diet-induced obesity in adipose tissue and pancreatic islets (Pejnovic et al. 2013)
*Lum*	May represent a functional link between the extracellular matrix and metabolic syndrome (Wolff et al. 2019)
*Mmp12*	Modulates HFD-induced glomerular fibrogenesis and inflammation in obese mouse (Niu et al. 2016)
*Mmp14*	Participates in obesity pathogenesis (Chun et al. 2010)
*Mmp19*	Knockout mice develop diet-induced obesity (Pendás et al. 2004)
*Mmp2*	Gene polymorphism linked with obesity in Korean population (Han et al. 2008)
*Olfml2b*	Overexpressed in pancreatic adenocarcinoma (Johnson et al. 2006)
*Serpine2*	Downregulation by miRNA linked with diabetic nephropathy (Li et al. 2017)
*Sod3*	Overexpressed in chronic pancreatitis compared to the healthy pancreas (Chen et al. 2007)
*Tgfb1*	Overexpression leads to diabetic nephropathy in mice (Hathaway et al. 2015)
*Tgfb3*	Higher levels observed in cancer patients compared to healthy tissues (Hachim et al. 2018)
*Timp1*	Regulates adipogenesis in obesity (Meissburger et al. 2011)
*Tinagl1*	Upregulated in the highly metastatic tumor (Umeyama et al. 2014)
*Zg16*	Low expression observed in colorectal cancer (Chen et al. 2016)
*Downregulated in NZO vs. C3H*	
*Hapln1*	Differential expression in pancreatic islets between B6-ob/ob and NZO mice (Kluth et al. 2015)
*Lpl*	Low expression observed in patients with T2D and insulin resistance (Huang et al. 2013)
*Matn2*	Depending on cancer type, linked with suppression and promotion of tumor (Korpos et al. 2015)
*Npnt*	ECM remodeling of Sca1^high^ ASCs (Tokunaga et al. 2014)
*Spock3*	Enriched in human α-cells (Xin et al. 2016)

^a^References are listed in Supplementary File 10

### Pathway enrichment analysis of AS genes

**Fig. 4:**
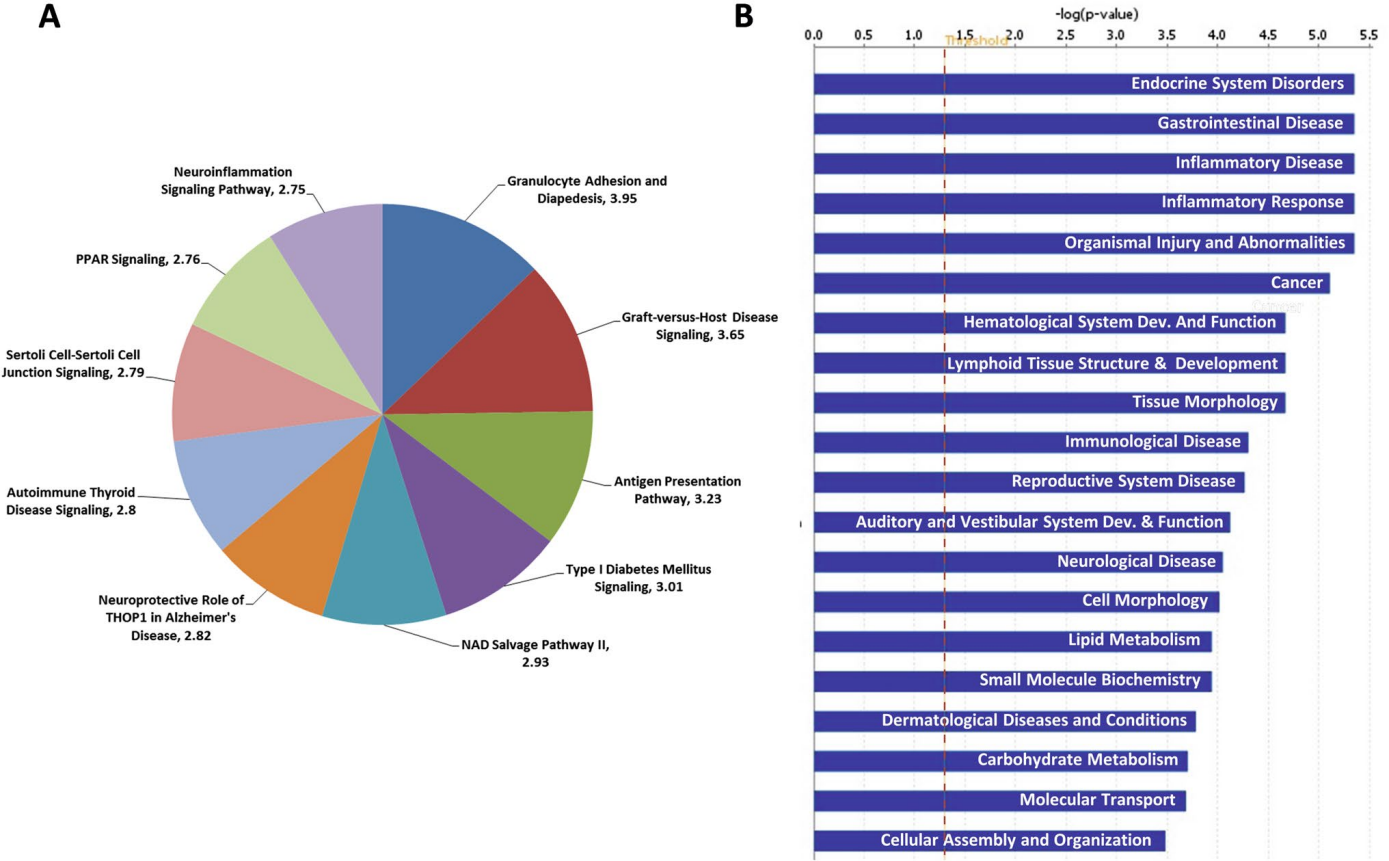
Ingenuity Pathway Analysis of alternatively spliced (AS) genes of pancreatic islets between C3H vs. NZO mice. **a** Top canonical pathways overrepresented in DE genes. Statistical significance is mentioned along with the pathway name as − log(*p*-value). **b** Top disease and function overrepresented by DE genes. The red dotted line represents the significance threshold (*p* < 0.05). Only top disease and function categories are mentioned

In the case of AS genes, 52 Ingenuity canonical pathways were overrepresented significantly (Supplementary File 6). Figure 4a represents the top canonical pathways in the list enriched with AS genes. These include granulocyte adhesion and diapedesis (*p-*value = 1.13E−04), graft-versus-host disease signaling (*p-*value = 2.24E−04), antigen presentation pathway (*p-*value = 5.94E−04), type I diabetes mellitus signaling (*p-*value = 9.66E−04), and NAD salvage pathway II (*p-*value = 1.17E−03).

Our bioinformatics analysis of the AS genes further revealed 79 categories related to disease and function (Supplementary File 7). As seen in Fig. 4b, the top categories in disease and function are endocrine system disorders, gastrointestinal disease, inflammatory disease, inflammatory response, and organismal injury and abnormalities.

### PPAR signaling and adipogenesis pathway are both enriched for DE and AS genes

**Fig. 5:**
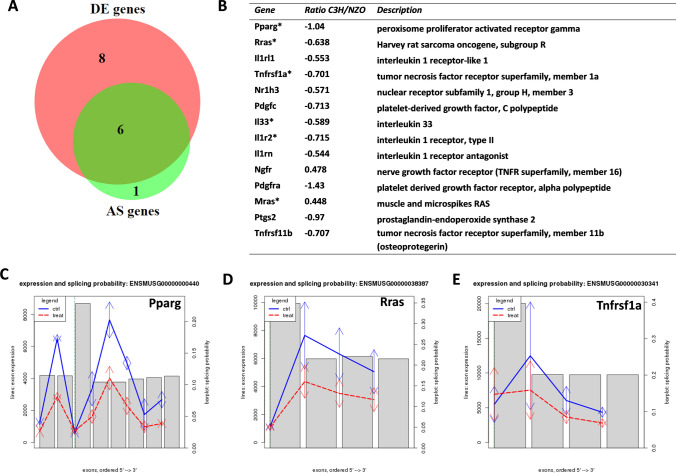
DE and AS genes related to PPAR signaling pathway. **a** Venn diagram to represent the DE and AS genes of the pathway. Out of 15 genes, 8 were DE while 6 genes were both DE as well as AS. One gene (*Nrip1*) belonged exclusively to the AS category. **b** List of DE genes related to PPAR signaling pathway. List prepared using Ingenuity Pathway Analysis. Genes marked with * were also reported to be alternatively spliced. Alternative splicing events are shown in the case of *Pparg* (**c**), *Rras* (**d**) and *Tnfrsf1a* (**e**). Solid lines (*y*-axis, left scale) show the exon expressions ordered by genomic position (*x*-axis). Blue line: NZO, red line C3H mice. Bars (*y*-axis, right scale) indicate the splicing probability values of the respective exons judged by the deviation of the exon expression log2-ratio (C3H vs NZO) and the median log2-ratio over all exons using ARH

In our study, PPAR signaling as well as adipogenesis pathway, two of the most studied metabolic pathways, were overrepresented in NZO compared to C3H for both DE and AS genes. Both pathways have been shown to play key roles in the regulation of insulin secretion and apoptosis in the pancreatic β-cell (Gerst et al. 2019; Gupta et al. 2010). Figure 5a represents all DE and AS genes related to PPAR signaling pathway obtained by IPA. Out of 15 genes, 8 genes were only DE, while 6 genes (*Il33*, *Pparg*, *Il1r2, Rras*, *Tnfrsf1a*, and *Mras*) were DE as well as AS between the pancreatic islets from C3H vs. NZO (Fig. 5b). The gene *Nrip1* was only alternatively spliced. The AS events in *Pparg*, *Rras*, and *Tnfrsf1a* are shown in Fig. 5c–e.

**Fig. 6:**
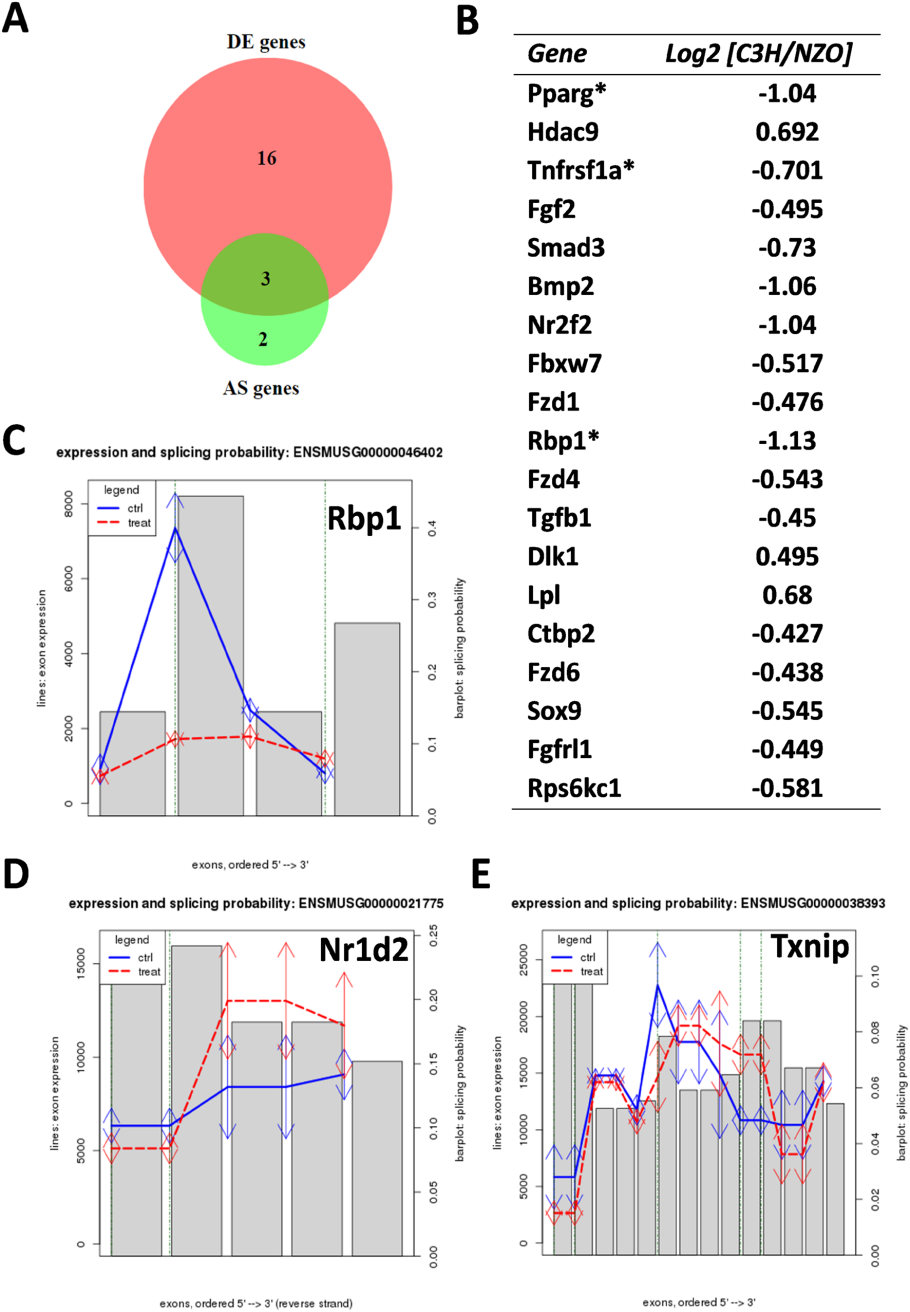
DE and AS genes related to adipogenesis pathway. **a** Venn diagram to represent the DE and AS genes of the pathway. Out of 21 genes, 16 were DE while three were both DE as well as AS. Two genes (*Nr1d2* and *Txnip*) belonged exclusively to the AS category. **b** List of DE genes related to Adipogenesis pathway. Genes marked with * were also reported to be alternatively spliced. Figure **c**–**e** represent the alternative splicing events in *Rbp1*, *Nr1d2*, and *Txnip*, respectively. Solid lines (*y*-axis, left scale) show the exon expressions ordered by genomic position (*x*-axis). Blue line: NZO, red line C3H mice. Bars (*y*-axis, right scale) indicate the splicing probability values of the respective exons judged by the deviation of the exon expression log2-ratio (C3H vs NZO) and the median log2-ratio over all exons using ARH

In context with the adipogenesis pathway, we identified 16 DE genes, among those three genes (*Pparg*, *Tnfrsf1a*, and *Rbp1*) which further revealed to be AS (Fig. 6a). The two genes *Nr1d2* and *Txnip* were only alternatively spliced. The AS events in *Rbp1*, *Nr1d2*, and *Txnip* are shown in Fig. 6c–e.

### Localization of DE and AS genes in T2D-associated QTL detected in the N_2_(NZOxC3H) population

We further investigated whether any of the top DE (log2 C3H/NZO > 1.2, < − 1.5) or/and top 20 AS genes localizes in a T2D-associated QTL that we reported recently in our N_2_(NZOxC3H) population (Schallschmidt et al. 2018) and, thus, represent potential candidates for the respective loci (Table 5). Our N_2_(NZOxC3H) population consists of 329 males which were phenotyped for several T2D-associated traits, including blood glucose and plasma insulin levels. Subsequent whole-genome linkage scans on the N_2_ mice revealed major new T2D QTL on chromosomes 4, 7, and 15. Several of our DE or/and AS genes are located in these loci. The DE gene *Gjb4* localizes within a QTL for blood glucose on distal Chr.4, designated as *Nbg4d* (*NZO blood glucose on distal Chr. 4*). The blood glucose and insulin-associated QTL on proximal Chr.7 (*Nbg7p*, *NZO blood glucose on proximal Chr. 7*) harbors six DE (*Sync*, *Klk1, Klk1b4*, *Klk1b5, Klk1b22* and *Atp4a*) and one AS (*Zfp715*) gene, whereas the distal blood glucose QTL on the same Chr. (*Nbg7d*, *NZO blood glucose on distal Chr. 7*) contains one DE (*Aldh1a3*) and one AS (*Trpm1*) gene. Furthermore, for *Nbg15p* (*NZO blood glucose on proximal Chr. 15*), we identified *Csfrb2* being DE and *Lgals2* being DE as well as AS in islets from NZO vs C3H mice.

**Table 5:** DE and AS genes located in T2D QTL identified in the N_2_(NZOxC3H) population

QTL name	Chr. (position closest SNP marker)	Phenotype(s)	DE genes	AS genes
*Nbg4d*	4 (119 Mb)	BG, plasma insulin, pancreas insulin	*Gjb4*	/
*Nbg7p*	7 (37.3 Mb)	BG, lean mass, plasma insulin	*Sycn, Atp4a, Klk1b4, Klk1b5, Klk1, Klk1b22,*	*Zfp715*
*Nbg7d*	7 (76.7 Mb)	BG	*Aldh1a3*	*Trpm1*
*Nbg15p*	15 (63.3 Mb)	BG	*Csf2rb2, Lgals2*	*Lgals2*

*Chr.* Chr., *BG* blood glucose, *QTL* quantitative trait locus

## Discussion

Both differential gene expression (DE) and alternative splicing (AS) may have profound effects on cellular homeostasis (Paronetto et al. 2016). In the present study we investigated DE and AS in pancreatic islets at the early phase of diabetes development using two inbred mouse strains that differ markedly in diabetes susceptibility. Many of the genes revealed from our analysis, such as *Aldh1a3* and *Cd36*, have been associated with T2D-related phenotypes recently, validating our approach. The described roles in context with β-cell function and/or T2D susceptibility of our strongest DE and AS genes are listed in Supplementary File 9. In addition, we present new genes, pathways and disorders that have not been described in context with T2D development before and thus provide novel potential targets for the research of the complex disease.

Numerous studies have demonstrated the heritability of T2D susceptibility in both humans and mice (Fuchsberger et al. 2016; Joost and Schurmann 2014). The NZO mouse strain has been established as a polygenic model for T2D, displaying early onset obesity, and consecutive β-cell loss, both strictly dependent on the diet (Chadt et al. 2008; Dreja et al. 2010; Jurgens et al. 2007). On a high-caloric diet rich in fat and carbohydrates, NZO mice develop hyperinsulinemia, followed by hyperglycemia starting at week 6, progressing into hypoinsulinemia, severe ketosis and eventually death. In contrast, C3H mice are widely protected from T2D on a HFD (Schallschmidt et al. 2018). To investigate the regulatory network prior β-cell destruction, we analyzed the transcriptome of pancreatic islets of NZO mice in the hyperglycemic, hyperinsulinemic (prediabetic) state and compared the data with expression profiles in islets derived from normoglycemic, normoinsulinemic C3H mice. Interestingly, NZO mice fed a high-fat carbohydrate-free diet remain normoglycaemic, whereas the same mice fed a carbohydrate-containing high-fat diet develop severe diabetes (Jurgens et al. 2007), indicating that glucotoxiciy is the predominant cause for β-cell failure.

Our microarray analysis revealed > 1200 DE islet genes between NZO and C3H. This high number of DE genes may reflect an adaptive attempt of the islet to counteract for the HFD challenge, but further strain specific expression cannot be excluded. We identified several members of the regenerating protein family (*Reg1*, *Reg2*, *Reg3b* and *Reg3g*) among the top genes, upregulated in NZO. The Reg proteins are secretory proteins that are involved in the proliferation and differentiation of different cells types. In context with T2D, the proteins are described to have protective impact on β-cell function (Calderari et al. 2014; Li et al. 2017; Xiong et al. 2011). *Reg1* is known to play an important role in the proliferation of acinar and islet cells (Zenilman et al. 1996) and is reported to be expressed exclusively during the proliferation in β-cells after damage (Parikh et al. 2012). The gene *Aldh1a3* is an established marker for β-cell dedifferentiation in rodents (Burke et al. 2017) and humans (Cinti et al. 2016). In line with these observations, the gene was threefold upregulated in the prediabetic NZO islets. Surprisingly, we identified several genes upregulated in NZO, which are described to be expressed exclusively in the exocrine pancreas, such as several cationic trypsinogen genes (*Try3* (*Prss3*), *Try4* and *Try5*) and the trypsin inhibitor *Spink3*, the moue homolog of human SPINK1. Similar as in our study, the inclusion of exocrine cells (“exocrinization”) in diabetic islets has already been observed before in NZO and other diabetic mice (Junger et al. 2002; Stoehr et al. 2000). However, whereas Junger et al. hypothesize that this exocrinization phenomena causes a loss of structure of the islets, Stoehr and his team rather believe that it appears secondary to apoptosis. Interestingly, co-localization of exocrine and endocrine markers has also been reported in the pancreas from human patients with T2D and is suggested to reflect transdifferentiation of acinar to β-cells or vice versa (Masini et al. 2017). The exact biological meaning of the observed expression of acinar genes in our islets from NZO remains to be elucidated.

Moreover, several genes of the kallikrein family (*Klk1*, *Klk1b4*, *Klk1b5*, and *Klk1b22*) revealed to be DE between NZO and C3H. The different kallikrein genes encode for serine proteases that are essential to many biological processes, including inflammation and the organization of the extracellular matrix (Lawrence et al. 2010). From all genes detected in our microarray analysis, *Klk1b22* represents the most downregulated gene in NZO islets. The only known substrate of *Klk1b22* is bradykinin. Bradykinin was already shown to have beneficial effects on β-cell survival in vitro (Xu et al. 2012), but the underlying mechanism remains to be elucidated. *Klk1b22* and the other kallikrein members DE in our study reside in the confidence interval of our strongest T2D QTL on proximal Chr.7 (designated *Nbg7p*). We recently nominated the two genes *Pop4* and *Atp4a* as potential causal gene variants for the QTL (Schallschmidt et al. 2018), but a causal relationship with the kallikrein locus should be considered as well. *Gjb4*, another gene upregulated in NZO, is localized in a T2D QTL on distal chromosome 4 (designated *Nbg4d*), associated with improved glycemia in NZO-allele carriers (Schallschmidt et al. 2018). Recently, overexpression of *Gjb4* in primary islets was shown to impair β-cell proliferation and insulin secretion (Gassler et al. 2020), indicating that *Gjb4* functions as a risk gene in T2D development and may thus contribute to the high T2D susceptibility in NZO. However, a causal relationship with our QTL which is associated with a protection from T2D in NZO-allele carriers, is rather unlikely.

Moreover, 14 genes from our DE list are associated with mitochondrial function (Table 2). Mitochondrial alterations are closely connected with the development of obesity, insulin resistance, and T2D (Bournat and Brown 2010; Patti and Corvera 2010). In obese individuals, alteration in the expression of nuclear-encoded mitochondrial genes was observed (Wilson-Fritch et al. 2004). Several mitochondrial encoded tRNAs (*mt-Tf*, *mt-Ti*, *mt-Tl2*, *mt-Tm*, *mt-Tq*) were downregulated in NZO islets. There is increasing evidence that mutations in the tRNA, which mostly occur in mitochondrial tRNAs, are associated with many complex human diseases (Wallace 2015), including T2D (Zhou et al. 2019). The observed downregulation of the five different mitochondrial tRNAs might contribute to hypersecretion of insulin and subsequent failure of the NZO islets.

Alternative splicing plays an essential role in regulating critical physiological pathways and leads to the generation of the complex proteome in the cell. To our knowledge, this study presents the first survey of alternative splicing in prediabetic islets at the global transcriptome level. Our analysis revealed 436 AS genes, among those 206 also DE between NZO and C3H. Several genes were implicated previously to play roles in diabetes-related phenotypes. *Apobec1*, an apolipoprotein B mRNA editing enzyme, tops the AS list with the highest ARH score. Alternatively spliced transcripts of *Apobec1* have been reported in different tissues in mice (Nakamuta et al. 1995). Target transcripts edited by APOBEC1 include the Alzheimer's amyloid precursor protein (*App*) which has been identified as candidate regulator of insulin secretion (Tu et al. 2012).

Alternative splicing of *Kcnip3*, a gene associated with diabetic retinopathy (Chavira-Suarez et al. 2011), is assumed to contribute to the diverse functions of the KCNIP proteins in the cells (Pruunsild and Timmusk 2005). Another gene from our AS list, *Nrip1*, codes for a corepressor for nuclear receptors that regulate the expression of metabolic genes involved in glucose and lipid metabolism (Nichol et al. 2006). *Nrip1* is known to be involved in the development of obesity and diabetes but not with the development of insulin resistance (Skrypnik et al. 2017). Multiple promoters and noncoding exons participate in alternative splicing giving rise to variants that are differentially utilized. Recently, overexpression of *Lefty1*, another gene from our AS list, was shown to increase proliferation in primary mouse islets (Kluth et al. 2015), whereas the DNA repair protein RPS3 is reported to be overexpressed in the presence of cellular stress (Shin et al. 2004). Thus, it seems that AS in NZO mostly appears as an adaptation process allowing to cope with increased metabolic stress.

Three genes (*Zfp715*, *Trpm1*, *Lgals2*) from our AS list, all of them with unknown functions in islets, are located in a T2D QTL identified in our N2 population. The gene *Lgals2* is the only QTL-associated gene that overlapped between our AS and DE gene list. Interestingly, a sequence variant in *Lgals2* has been linked with altered plasma insulin and glucose levels in humans (Christensen et al. 2006), underscoring its potential relevance in human T2D pathogenesis. The closely related gene *Lgals3* has already been shown to protect from HFD-induced obesity in mice (Pejnovic et al. 2013), but functional evidence for *Lgals2* in context with T2D development is still missing.

A recent study of T2D islets showed dysregulated splicing for ~ 25% of splicing events, where genes involved in mRNA processing and expression were enriched (Jeffery et al. 2019). According to our pathway analysis a large number of canonical pathways in islets are affected following HFD treatment of the two mouse strains. Ingenuity software demonstrated overrepresentation of 149 and 52 canonical pathways related to DE and AS genes, respectively.

As expected, many pathways and disorders associated with glucose metabolism were overrepresented for both DE and AS genes. The LXR/RXR activation, which is known to be regulated by lipophilic small molecules such as steroid hormones, thyroid hormones, and vitamin D3, was the most affected pathway associated with DE genes. LXR activation in pancreatic β-cells has been shown to increase insulin secretion and insulin biosynthesis by modulation of the glucose and lipid metabolism (Efanov et al. 2004). Inflammation-related pathways were overrepresented in both DE and AS genes. HFD-induced inflammation has been extensively reported in several studies (Park et al. 2014; Stemmer et al. 2012). While only about half of the pathways related to AS overlapped with the DE pathways, almost all (65 of 68) of the categories related to disease & function for the AS genes overlapped with the DE categories. Thus, it seems that DE and AS have unique roles in different pathways, which together contribute to the pathogenesis of the disease phenotype.

Alternative splicing is an important control mechanism for cell phenotype and is often deregulated in disease (Stevens and Oltean 2016). The exact functional implications of AS in T2D is, however, not well understood. Protein variants produced by AS may differ in size and domain organization, putative interaction with low molecular ligands and/or other proteins, secondary modifications as well as in biological half times. Furthermore, genetic mutations that occur in AS exons may lead to tissue-specific loss of function, as exemplified by a common muscle-specific *TBC1D4* p.Arg684T variant, which accounts for more than 10% of all diabetes cases in Greenlandic and other Arctic populations (Moltke et al. 2014). Splicing alterations during the development of type 1 and type 2 diabetes is also suggested to be linked to pancreatic β-cell demise (Cnop et al. 2014; Eizirik et al. 2012). Evidence of AS in β‐cell function and failure has been reviewed recently (Alvelos et al. 2018). Further research is necessary to evaluate the functional impact of AS in insulin secretion and islet cell survival.

Interestingly, the extracellular matrix revealed to be one of the most affected cellular components related to DE genes in our GO analysis. Molecules present in the ECM not only provide structural support but also play an essential role in the molecular signaling and repair of many organ structures like the pancreas. These ECM molecules play a critical role in multiple aspects of islet physiology and regulate β-cell survival and insulin production (Llacua et al. 2018). Alteration in the ECM was reported to cause obesity-associated insulin resistance (Lin et al. 2016). HFD is known to alter the gene expression leading to ECM remodeling (Pincu et al. 2016). Most of the ECM-associated DE genes were upregulated in NZO islets, indicating that these genes may function in the stabilization and repair of the stressed ECM due to HFD-induced gluco- and lipotoxicity. In line with this hypothesis, several metallopeptidases of the *Adamts* (*Adamts1*, *Adamts4*, *Adamts5*, and *Adamts9*) and *Mmp* gene family (*Mmp2*, *Mmp12*, *Mmp14*, and *Mmp19*) with well described to function in ECM remodeling (Cui et al. 2017; Kelwick et al. 2015), were upregulated in NZO. Whereas *Mmp* gene family members, in particular *Mmp9*, were already shown to be essential for normal β-cell function (Christoffersson et al. 2015), members of the *Adamts* gene family have not been described in context with β-cell function, yet. Our analysis revealed several further novel potential players for β-cell function.

Interestingly, *PPARG* and adipogenesis pathways, two well-described metabolic pathways, were overrepresented in our DE and AS genes. PPARG signaling is reported to regulate directly key β-cell genes involved in glucose sensing, insulin secretion and insulin gene transcription (Gupta et al. 2010), whereas the secretome from pancreatic adipocytes is assumed to influence insulin secretion and β-cell survival (Gerst et al. 2019). For both pathways, the associated DE genes were almost all upregulated in NZO islets. Increased adipogenesis signaling likely mirrors ectopic fat accumulation in NZO islets, which is a well-known trigger of β-cell failure. Similarly, increased PPARG signaling might reflect increased glucose and fat metabolism in NZO to compensate for nutritional overload. It seems likely that upregulation and AS of genes related to PPARG signaling represents an adaptive mechanism to counteract for HFD-induced cellular stress.

## Conclusion

Our study provides novel target genes and pathways that may explain the high T2D susceptibility observed in NZO and potentially in humans. Moreover, our data show that AS and DE in large parts seem to act in different pathways, suggesting that AS takes a unique role in T2D development. This information may contribute to our growing understanding of the pathomechanisms that lead to β-cell failure and provide novel potential targets for therapeutic strategies.

## Supplementary Information

Below is the link to the electronic supplementary material.

Supplementary file1 (XLSX 228 kb)

Supplementary file2 (XLSX 986 kb)

Supplementary file3 (XLSX 19 kb)

Supplementary file4 (XLSX 43 kb)

Supplementary file5 (XLSX 146 kb)

Supplementary file6 (XLSX 11 kb)

Supplementary file7 (XLSX 17 kb)

Supplementary file8 (PDF 585 kb)

Supplementary file9 (PDF 678 kb)

Supplementary file10 (PDF 418 kb)

## Data Availability

All Supplementary Files are available on the following link: http://87.193.3.78:8080/share.cgi?ssid=0V2Gjv1. Supplementary Files 1–10 contain the following information: 1, all DE genes; 2, all AS genes; 3, enriched pathways of DE genes; 4, diseases and functions of DE genes; 5, cell components of DE genes; 6: enriched pathways of AS genes; 7, diseases and functions of AS genes; 8, qPCR analysis of selected DE genes; 9, DE and AS genes associated with pancreatic β-cell function and /or T2D development; 10, references referring to Table 4. Microarray data are available under accession number GSE117553.
