# Supplementary material for: Alternative exon splicing and differential expression in pancreatic islets reveals candidate genes and pathways implicated in early diabetes development
Source: Mamm Genome. 2021 Apr 20;32(3):153–72. doi: 10.1007/s00335-021-09869-1 (PMC8128753; doi:10.1007/s00335-021-09869-1)
Supplement: Supplementary file 8 — Supplementary file8 (PDF 585 kb) [file 335_2021_9869_MOESM8_ESM.pdf]

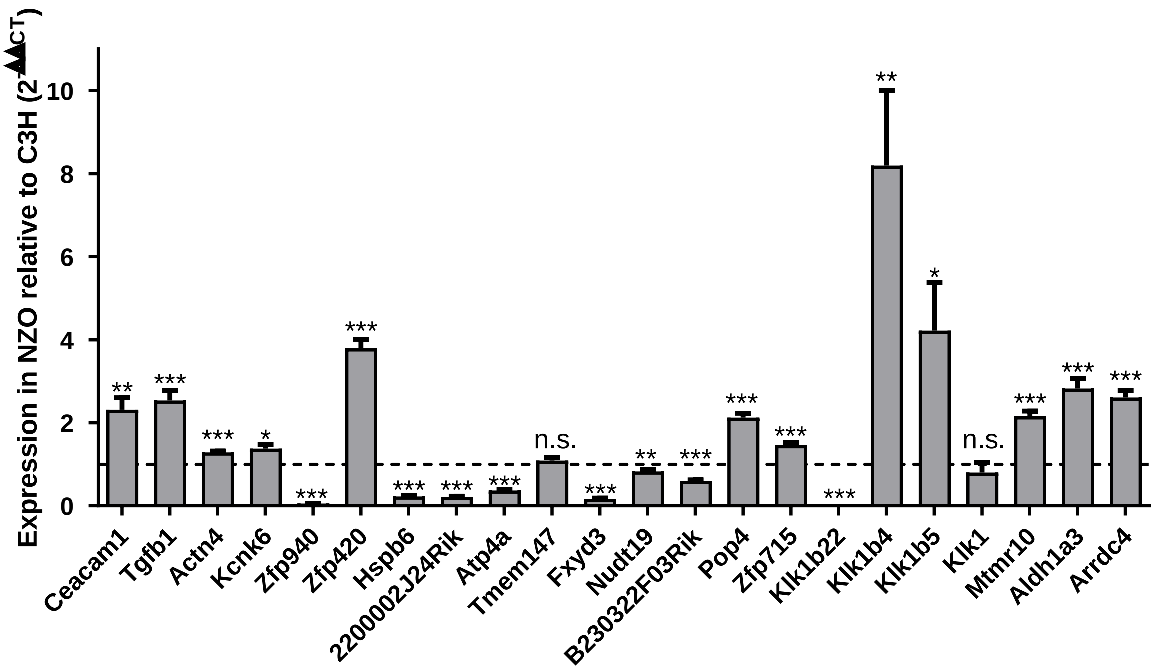

Supplementary File 8 Validation of differential gene expression in the pancreatic islets from NZO and C3H by quantitative real-time PCR. The expression levels in pancreatic islets were normalized to C3H as indicated by the dotted line. TATA box binding protein (*Tbp*) was used as an endogenous control. Out of 22 genes that revealed significant differential gene expression in the microarray analysis, 20 were validated. Data represent mean values ± SEM from eight mice. Statistical differences between both strains were calculated by unpaired t-test. * P, 0.05, ** P, 0.01 and *** P, 0.001

Primers used for quantitative real-time PCR. Primer sequences were synthesized by Eurogentec (Seraing, Belgium). Primers were dissolved in nuclease free water to reach a final concentration of 10 nM. Fwd, Forward; Rev, Reverse.

**Target**	**Sequence 5'→3'**
Tbp	Fwd: GCGGCACTGCCCATTTATTT<br>Rev: GGCGGAATGTATCTGGCACA
Ceacam1	Fwd: AGGGTGACGACTCCGTATCA<br>Rev: GGGGTGTCCGGACCATAGAT
Tgfb1	Fwd: GTTCACGGGATACTCCAGGT<br>Rev: CCTCTGGACCTGGAAGGAGTTA
Kcnk6	Fwd: TGCTTGTCACAGCGTACCTC<br>Rev: GATCGGGCAGCAAGATGAGT
Zfp940	Fwd: GGAAGCCAGTTAGAAGCCGT<br>Rev: GAGCCCGAGAAAGATTGGGA
Zfp420	Fwd: TCACGAGAGGGTTGTGTCTGG<br>Rev: ACATCCCTGATTTCTCAAACTTCC
Hspb6	Fwd: CTCTTTGACCAGCGTTTCGG<br>Rev: CATCCAGCAGCACGGAAAAA
2200002J24Rik	Fwd: CCCCAGCGAAGGATTACCTC<br>Rev: GCTTCCCGGGTCTTAAGGG
Atp4a	Fwd: GGTTAATAGGAAGGATGCCCG<br>Rev: CAGTCGCTGACAACTCTCCA
Tmem147	Fwd: TGTCATGGAGACCTTCGTCC<br>Rev: GGTTCGCCAACACCTGAGAT
Fxyd3	Fwd: ACAGAAACCCAGTCACCGCC<br>Rev: AGTTCAAGCCCACCTTCAGAG
Nudt19	Fwd: CTACCAGTGGTTGTCCCCAT<br>Rev: TGATGGGCGATCCGAACAAA
B230322F03Rik	Fwd: GTCAGGGTCATTCTCGCCC<br>Rev: CCTATACAGGCAACAGGCCA
Pop4	Fwd: CGATGTGCAGGAGCTGGGAA<br>Rev: TGTCAAAGAGCCGCATGTCT
Zfp715	Fwd: TCGAGTGTTACTTCGCTGGC<br>Rev: AGAAGGGCTAGCTCACACTG
Klk1b22	Fwd: TGTGTCCATCAAGCTCAATCC<br>Rev: GCCTCCTGAGTCTCCCTTACA
Klk1b4	Fwd: CGATGTGCAGGAGCTGGGAA<br>Rev: TGTCAAAGAGCCGCATGTCT
Klk1b5	Fwd: ACCCGTCATATACGAACCCG<br>Rev: ACCATCACAGATCAGTGGGC
Klk1	Fwd: AAGACACTTGTGCGGGTGAC<br>Rev: ACTCAGTCATTTTCAGCCATAGTTT
Mtmr10	Fwd: AGTCCTAGGTCCCAACCAGA<br>Rev: TTGCAAGGCATACCTTCTTGG
Aldh1a3	Fwd: GAGCGATCCTGGCTACTCTG<br>Rev: TCATCTGTGGGGATGGTCCT
Arrdc4	Fwd: GCTGGAGCTGCCTTTGGTTA<br>Rev: TAATTTGGTGGTGCTTCAGGC

TaqMan probes used for quantitative real-time PCR. TaqMan probes were purchased from Thermo Fisher Scientific (Darmstadt, Germany)

**Target**	**Assay ID**
Actn4	Mm00502489_m1
Zfp719	Mm01197328_m1
