# Supplementary material for: Alternative exon splicing and differential expression in pancreatic islets reveals candidate genes and pathways implicated in early diabetes development
Source: Mamm Genome. 2021 Apr 20;32(3):153–72. doi: 10.1007/s00335-021-09869-1 (PMC8128753; doi:10.1007/s00335-021-09869-1)
Supplement: Supplementary file 9 — Supplementary file9 (PDF 678 kb) [file 335_2021_9869_MOESM9_ESM.pdf]

Supplementary File 9 List of DE and AS genes[1] with known role in pancreatic β-cell function and /or type 2 diabetes development

Gene(s)	Described role in type 2 diabetes development and / or β-cell function (reference)
**Upregulated in NZO**	
Prss3	Genetic variant associated with human T2D in humans (Bandesh and Bharadwaj 2020)
Reg1, Reg2, Reg3b, Reg3g	Protective impact on β-cell function (Chen, et al. 2019), Upregulated in islets from diabetic Goto-Kakizaki rats (Calderari, et al. 2014)
Aldh1a3	Marker for β-cell dedifferentiation in rodents and humans (Burke, et al. 2017; Cinti, et al. 2016)
Lgals2	Linked with altered plasma insulin and glucose levels in humans (Christensen, et al. 2006); forms a complex with cationic amino acid transporter in pancreatic β-cells, thereby regulating insulin secretion (Maeda, et al. 2020)
Cela3b, Cela2a, Cpa2	Protein abundance upregulated in islets from Zucker Diabetic Fatty rats (Han, et al. 2011)
Cpb1	Regulates β-cell proliferation after phosphorylation (Yu, et al. 2017)
Cck	Promotes β-cell proliferation in mouse and human islets (Lavine, et al. 2008; Schmid, et al. 1989)
Gjb4	Impairs cell proliferation and insulin secretion in primary mouse islets (Gassler, et al. 2020)
Cd36	Contributes to pancreatic β-cell dysfunction and damage (Moon, et al. 2020; Nagao, et al. 2020)
Serpinb1a	Promotes pancreatic β-Cell Proliferation (El Ouaamari, et al. 2016)
Ctsh	Regulates β-cell function and disease progression in type 1 diabetes (Floyel, et al. 2014)
Clps	SNPs associated with a reduced insulin secretion (Weyrich, et al. 2009)
**Upregulated in C3H**	
Atp4a	Alters glucose-stimulated insulin secretion in MIN6 cells (Schallschmidt, et al. 2018)
Angptl7	Circulating cytokine, elevated levels in T2D patients and mouse models, promotes insulin resistance (Xu, et al. 2020)
Vip	Enhances glucose-stimulated insulin secretion from mouse β-cells (Kato, et al. 1994)
Galr1	Binds galanin, which regulates insulin release in several species (Ahrén and Lindskog 1992; Boyle, et al. 1994)
Calb1	Regulates Ca^{2+} currents through VDCC, inhibits GSIS in pancreatic β-cells (Lee, et al. 2006; Parkash, et al. 2002)
Rims1	Implicated in insulin exocytosis in INS-1E cells (Iezzi, et al. 2000)
Slc2a2	Major glucose transporter in rodent islet β-cells (Thorens 2015)
Gad1	Contributes to compensatory insulin secretion in aged mouse pancreatic islets (Cho, et al. 2019)
**AS between NZO and C3H**	
Apobec1	Downregulated in diabetic Goto-Kakizaki rat islets (Ghanaat-Pour, et al. 2007)
Nrip1	Associated with subclinical inflammation in type 2 diabetic patients (Xue, et al. 2013), promotes glucolipotoxicity-induced damage in MIN6 cells (Xue, et al. 2018)
Lefty1	Increases pancreatic β-cell proliferation in mice (Kluth, et al. 2015)

[1]Only the top differentially expressed genes (Tables 1A and 1B) as well as the top AS genes (Table 3) were selected for the literature research in PubMed (https://pubmed.ncbi.nlm.nih.gov/)

Literature

Ahrén, B., and S. Lindskog
1992 Galanin and the regulation of islet hormone secretion. Int J Pancreatol 11(3):147-60.
Bandesh, K., and D. Bharadwaj
2020 Genetic variants entail type 2 diabetes as an innate immune disorder. Biochim Biophys Acta Proteins Proteom 1868(9):140458.
Boyle, M. R., et al.
1994 Canine galanin: sequence, expression and pancreatic effects. Regul Pept 50(1):1-11.
Burke, S. J., et al.
2017 db/db Mice Exhibit Features of Human Type 2 Diabetes That Are Not Present in Weight-Matched C57BL/6J Mice Fed a Western Diet. J Diabetes Res 2017:8503754.
Calderari, S., et al.
2014 Regenerating 1 and 3b gene expression in the pancreas of type 2 diabetic Goto-Kakizaki (GK) rats. PLoS One 9(2):e90045.
Chen, Z., S. Downing, and E. S. Tzanakakis
2019 Four Decades After the Discovery of Regenerating Islet-Derived (Reg) Proteins: Current Understanding and Challenges. Front Cell Dev Biol 7:235.
Cho, J. H., et al.
2019 Glutamate decarboxylase 67 contributes to compensatory insulin secretion in aged pancreatic islets. Islets 11(2):33-43.
Christensen, M. B., et al.
2006 Genotype of galectin 2 (LGALS2) is associated with insulin-glucose profile in the British Women's Heart and Health Study. Diabetologia 49(4):673-7.
Cinti, F., et al.
2016 Evidence of beta-Cell Dedifferentiation in Human Type 2 Diabetes. J Clin Endocrinol Metab 101(3):1044-54.
Floyel, T., et al.
2014 CTSH regulates beta-cell function and disease progression in newly diagnosed type 1 diabetes patients. Proc Natl Acad Sci U S A 111(28):10305-10.
Gassler, A., et al.
2020 Overexpression of Gjb4 impairs cell proliferation and insulin secretion in primary islet cells. Mol Metab 41:101042.
Ghanaat-Pour, H., et al.
2007 Global expression profiling of glucose-regulated genes in pancreatic islets of spontaneously diabetic Goto-Kakizaki rats. J Mol Endocrinol 39(2):135-50.
Han, D., et al.
2011 Detection of differential proteomes associated with the development of type 2 diabetes in the Zucker rat model using the iTRAQ technique. J Proteome Res 10(2):564-77.
Iezzi, M., R. Regazzi, and C. B. Wollheim
2000 The Rab3-interacting molecule RIM is expressed in pancreatic beta-cells and is implicated in insulin exocytosis. FEBS Lett 474(1):66-70.
Kato, I., et al.
1994 Transgenic mice overexpressing human vasoactive intestinal peptide (VIP) gene in pancreatic beta cells. Evidence for improved glucose tolerance and enhanced insulin secretion by VIP and PHM-27 in vivo. J Biol Chem 269(33):21223-8.
Kluth, O., et al.
2015 Identification of Four Mouse Diabetes Candidate Genes Altering beta-Cell Proliferation. PLoS Genet 11(9):e1005506.
Lavine, J. A., et al.
2008 Overexpression of pre-pro-cholecystokinin stimulates beta-cell proliferation in mouse and human islets with retention of islet function. Mol Endocrinol 22(12):2716-28.
Lee, D., et al.

2006 Calbindin-D28k decreases L-type calcium channel activity and modulates intracellular calcium homeostasis in response to K+ depolarization in a rat beta cell line RINr1046-38. Cell Calcium 39(6):475-485.

Maeda, K., et al.

2020 Galectin-lattice sustains function of cationic amino acid transporter and insulin secretion of pancreatic beta cells. J Biochem 167(6):587-596.

Moon, J. S., et al.

2020 The Role of CD36 in Type 2 Diabetes Mellitus: beta-Cell Dysfunction and Beyond. Diabetes Metab J 44(2):222-233.

Nagao, M., et al.

2020 Potential Protection Against Type 2 Diabetes in Obesity Through Lower CD36 Expression and Improved Exocytosis in beta-Cells. Diabetes 69(6):1193-1205.

Parkash, J., et al.

2002 Intracellular calcium ion response to glucose in beta-cells of calbindin-D28k nullmutant mice and in betaHC13 cells overexpressing calbindin-D28k. Endocrine 18(3):221-9.

Schallschmidt, T., et al.

2018 Two Novel Candidate Genes for Insulin Secretion Identified by Comparative Genomics of Multiple Backcross Mouse Populations. Genetics 210(4):1527-1542.

Schmid, R., et al.

1989 Effect of CCK on insulin, glucagon, and pancreatic polypeptide levels in humans. Pancreas 4(6):653-61.

Thorens, B.

2015 GLUT2, glucose sensing and glucose homeostasis. Diabetologia 58(2):221-32.

Weyrich, P., et al.

2009 Genetic variability of procolipase associates with altered insulin secretion in non-diabetic Caucasians. Exp Clin Endocrinol Diabetes 117(2):83-7.

Xu, T., et al.

2020 Angptl7 promotes insulin resistance and type 2 diabetes mellitus by multiple mechanisms including SOCS3-mediated IRS1 degradation. FASEB J 34(10):13548-13560.

Xue, J., et al.

2013 RIP140 is associated with subclinical inflammation in type 2 diabetic patients. Exp Clin Endocrinol Diabetes 121(1):37-42.

Xue, L. J., et al.

2018 Up-regulation of receptor interaction protein 140 promotes glucolipotoxicity-induced damage in MIN6 cells. Cell Mol Biol (Noisy-le-grand) 64(4):39-45.

Yu, S. L., et al.

2017 Phosphorylation of carboxypeptidase B1 protein regulates beta-cell proliferation. Int J Mol Med 40(5):1397-1404.
