# Supplementary material for: Alternative exon splicing and differential expression in pancreatic islets reveals candidate genes and pathways implicated in early diabetes development
Source: Mamm Genome. 2021 Apr 20;32(3):153–72. doi: 10.1007/s00335-021-09869-1 (PMC8128753; doi:10.1007/s00335-021-09869-1)
Supplement: Supplementary file 10 — Supplementary file10 (PDF 418 kb) [file 335_2021_9869_MOESM10_ESM.pdf]

Supplementary File 10 References related to the function of all DE ECM-related genes in pancreatic islets from C3H vs. NZO (see Table 4 in the main manuscript)

Akhabir L, Sandford A (2010) Genetics of interleukin 1 receptor-like 1 in immune and inflammatory diseases. Curr Genomics 11, 591-606

Bauters D, Spincemaille P, Geys L, Cassiman D, Vermeersch P, Bedossa P, Scroyen I, Lijnen HR (2016) ADAMTS5 deficiency protects against non-alcoholic steatohepatitis in obesity. Liver Int 36, 1848-1859

Bogdanov P, Corraliza L, Villena JA, Carvalho AR, Garcia-Arumi J, Ramos D, Ruberte J, Simo R, Hernandez C (2014) The db/db mouse: a useful model for the study of diabetic retinal neurodegeneration. PLoS One 9, e97302

Chen R, Brentnall TA, Pan S, Cooke K, Moyes KW, Lane Z, Crispin DA, Goodlett DR, Aebersold R, Bronner MP (2007) Quantitative proteomics analysis reveals that proteins differentially expressed in chronic pancreatitis are also frequently involved in pancreatic cancer. Mol Cell Proteomics 6, 1331-1342

Chen X, Du P, She J, Cao L, Li Y, Xia H (2016) Loss of ZG16 is regulated by miR-196a and contributes to stemness and progression of colorectal cancer. Oncotarget 7, 86695-86703

Chun TH, Inoue M, Morisaki H, Yamanaka I, Miyamoto Y, Okamura T, Sato-Kusubata K, Weiss SJ (2010) Genetic link between obesity and MMP14-dependent adipogenic collagen turnover. Diabetes 59, 2484-2494

Daimon M, Oizumi T, Karasawa S, Kaino W, Takase K, Tada K, Jimbu Y, Wada K, Kameda W, Susa S, Muramatsu M, Kubota I, Kawata S, Kato T (2011) Association of the clusterin gene polymorphisms with type 2 diabetes mellitus. Metabolism 60, 815-822

Danussi C, Petrucco A, Wassermann B, Modica TM, Pivetta E, Del Bel Belluz L, Colombatti A, Spessotto P (2012) An EMILIN1-negative microenvironment promotes tumor cell proliferation and lymph node invasion. Cancer Prev Res (Phila) 5, 1131-1143

Hachim MY, Hachim IY, Dai M, Ali S, Lebrun JJ (2018) Differential expression of TGFbeta isoforms in breast cancer highlights different roles during breast cancer progression. Tumour Biol 40, 1010428317748254

Han DH, Kim SK, Kang S, Choe BK, Kim KS, Chung JH (2008) Matrix Metallopeptidase 2 Gene Polymorphism is Associated with Obesity in Korean Population. Korean J Physiol Pharmacol 12, 125-129

Hathaway CK, Gasim AM, Grant R, Chang AS, Kim HS, Madden VJ, Bagnell CR, Jr., Jennette JC, Smithies O, Kakoki M (2015) Low TGFbeta1 expression prevents and high expression exacerbates diabetic nephropathy in mice. Proc Natl Acad Sci U S A 112, 5815-5820

Ho MM, Yoganathan P, Chu KY, Karunakaran S, Johnson JD, Clee SM (2013) Diabetes genes identified by genome-wide association studies are regulated in mice by nutritional factors in metabolically relevant tissues and by glucose concentrations in islets. BMC Genet 14, 10

Huang Y, Li X, Wang M, Ning H, A L, Li Y, Sun C (2013) Lipoprotein lipase links vitamin D, insulin resistance, and type 2 diabetes: a cross-sectional epidemiological study. Cardiovasc Diabetol 12, 17

Johnson SK, Dennis RA, Barone GW, Lamps LW, Haun RS (2006) Differential expression of insulin-like growth factor binding protein-5 in pancreatic adenocarcinomas: identification using DNA microarray. Mol Carcinog 45, 814-827

Kluth O, Matzke D, Kamitz A, Jahnert M, Vogel H, Scherneck S, Schulze M, Staiger H, Machicao F, Haring HU, Joost HG, Schurmann A (2015) Identification of Four Mouse Diabetes Candidate Genes Altering beta-Cell Proliferation. PLoS Genet 11, e1005506

Koninger J, Giese NA, di Mola FF, Berberat P, Giese T, Esposito I, Bachem MG, Buchler MW, Friess H (2004) Overexpressed decorin in pancreatic cancer: potential tumor growth inhibition and attenuation of chemotherapeutic action. Clin Cancer Res 10, 4776-4783

Korpos E, Deak F, Kiss I (2015) Matrilin-2, an extracellular adaptor protein, is needed for the regeneration of muscle, nerve and other tissues. Neural Regen Res 10, 866-869

Kumar S, Chen M, Li Y, Wong FH, Thiam CW, Hossain MZ, Poh KK, Hirohata S, Ogawa H, Angeli V, Ge R (2016) Loss of ADAMTS4 reduces high fat diet-induced atherosclerosis and enhances plaque stability in ApoE(-/-) mice. Sci Rep 6, 31130

Le KA, Mahurkar S, Alderete TL, Hasson RE, Adam TC, Kim JS, Beale E, Xie C, Greenberg AS, Allayee H, Goran MI (2011) Subcutaneous adipose tissue macrophage infiltration is associated with hepatic and visceral fat deposition, hyperinsulinemia, and stimulation of NF-kappaB stress pathway. Diabetes 60, 2802-2809

Lee J, Song J, Kwon ES, Jo S, Kang MK, Kim YJ, Hwang Y, Bae H, Kang TH, Chang S, Cho HJ, Kim SC, Kim S, Koh SS (2016) CTHRC1 promotes angiogenesis by recruiting Tie2-expressing monocytes to pancreatic tumors. Exp Mol Med 48, e261

Li YB, Wu Q, Liu J, Fan YZ, Yu KF, Cai Y (2017) miR199a3p is involved in the pathogenesis and progression of diabetic neuropathy through downregulation of SerpinE2. Mol Med Rep 16, 2417-2424

Masui T, Hosotani R, Tsuji S, Miyamoto Y, Yasuda S, Ida J, Nakajima S, Kawaguchi M, Kobayashi H, Koizumi M, Toyoda E, Tulachan S, Arii S, Doi R, Imamura M (2001) Expression of METH-1 and METH-2 in pancreatic cancer. Clin Cancer Res 7, 3437-3443

Meissburger B, Stachorski L, Roder E, Rudofsky G, Wolfrum C (2011) Tissue inhibitor of matrix metalloproteinase 1 (TIMP1) controls adipogenesis in obesity in mice and in humans. Diabetologia 54, 1468-1479

Nadler ST, Stoehr JP, Schueler KL, Tanimoto G, Yandell BS, Attie AD (2000) The expression of adipogenic genes is decreased in obesity and diabetes mellitus. Proc Natl Acad Sci U S A 97, 11371-11376

Niu H, Li Y, Li H, Chi Y, Zhuang M, Zhang T, Liu M, Nie L (2016) Matrix metalloproteinase 12 modulates high-fat-diet induced glomerular fibrogenesis and inflammation in a mouse model of obesity. Sci Rep 6, 20171

Pejnovic NN, Pantic JM, Jovanovic IP, Radosavljevic GD, Milovanovic MZ, Nikolic IG, Zdravkovic NS, Djukic AL, Arsenijevic NN, Lukic ML (2013) Galectin-3 deficiency accelerates high-fat diet-induced obesity and amplifies inflammation in adipose tissue and pancreatic islets. Diabetes 62, 1932-1944

Pendas AM, Folgueras AR, Llano E, Caterina J, Frerard F, Rodriguez F, Astudillo A, Noel A, Birkedal-Hansen H, Lopez-Otin C (2004) Diet-induced obesity and reduced skin cancer susceptibility in matrix metalloproteinase 19-deficient mice. Mol Cell Biol 24, 5304-5313

Tokunaga M, Inoue M, Jiang Y, Barnes RH, 2nd, Buchner DA, Chun TH (2014) Fat depot-specific gene signature and ECM remodeling of Sca1(high) adipose-derived stem cells. Matrix Biol 36, 28-38

Umeyama H, Iwadate M, Taguchi YH (2014) TINAGL1 and B3GALNT1 are potential therapy target genes to suppress metastasis in non-small cell lung cancer. BMC Genomics 15 Suppl 9, S2

Wolff G, Taranko AE, Meln I, Weinmann J, Sijmonsma T, Lerch S, Heide D, Billeter AT, Tews D, Krunic D, Fischer-Posovszky P, Muller-Stich BP, Herzig S, Grimm D, Heikenwalder M, Kao WW, Vegiopoulos A (2019) Diet-dependent function of the extracellular matrix proteoglycan Lumican in obesity and glucose homeostasis. Mol Metab 19, 97-106

Wu QW, She HQ, Liang J, Huang YF, Yang QM, Yang QL, Zhang ZM (2012) Expression and clinical significance of extracellular matrix protein 1 and vascular endothelial growth factor-C in lymphatic metastasis of human breast cancer. BMC Cancer 12, 47

Xin Y, Kim J, Okamoto H, Ni M, Wei Y, Adler C, Murphy AJ, Yancopoulos GD, Lin C, Gromada J (2016) RNA Sequencing of Single Human Islet Cells Reveals Type 2 Diabetes Genes. Cell Metab 24, 608-615

Zhang L, Reidy SP, Nicholson TE, Lee HJ, Majdalawieh A, Webber C, Stewart BR, Dolphin P, Ro HS (2005) The role of AEBP1 in sex-specific diet-induced obesity. Mol Med 11, 39-47
